# VALIDATION OF THE PAGE-B SCORE AS A PROGNOSIS OF DEVELOPMENT TO HEPATOCELLULAR CARCINOMA (HCC) IN CHRONIC HEPATITIS B, IN THE BRAZILIAN POPULATION

**DOI:** 10.1590/S0004-2803.24612025-009

**Published:** 2025-12-01

**Authors:** Ingrid Laise Vivas SILVA, Liliane LINS-KUSTERER, Walter da SILVA, Jadson Dourado Costa FERNANDES, Sidelcina Rugieri PACHECO, Simone Muniz Carvalho Fernandes da CUNHA, Juan Miguel Villalobos SALCEDO, Luiz Felipe Monteiro DARZÉ, Raymundo PARANÁ, Maria Isabel SCHINONI

**Affiliations:** 1 Programa de Pós-Graduação em Processos Interativos de Órgãos e Sistemas - PPGPIOS-UFBA, Salvador, BA, Brasil.; 2 Hospital Universitário Professor Edgard Santos - HUPES, Salvador, BA, Brasil.; 3 Universidade Federal da Bahia - UFBA, Salvador, BA, Brasil.; 4 Centro de Medicina Tropical de Rondônia - CEMETRON, Porto Velho, RO, Brasil

**Keywords:** HBV, hepatocellular carcinoma, PAGE-B, risk score, HVB, carcinoma hepatocelular, page-B, escore de risco

## Abstract

**Context:**

Due to the potential risk for chronic and severe progression, hepatitis B virus (HBV) infection requires antiviral medications, such as Tenofovir (TDF) and Entecavir (ETV), to reduce the HBV viral load and prevent the risk of liver cirrhosis and hepatocellular carcinoma (HCC). The PAGE-B score is a simple and reliable tool for assessing the risk of developing HCC, although it has not yet been validated in Brazil.

**Objective:**

To validate the PAGE-B risk score for predicting HCC development in HBV carriers in Brazil. To analyze the association between the PAGE-B score and demographic, laboratory, and HBV treatment variables.

**Methods:**

An observational cohort. retrospective study. Study sample - 659 individuals with chronic HBV mono-infection treated with antivirals for at least 3 years at two reference centers in Brazil’s Northeast and Amazon regions. The PAGE-B score was used to analyze its association with sex, age, and platelet count, classifying each patient’s HCC risk as low, moderate, or high.

**Results:**

The mean PAGE-B score was 12.77±5.63. PAGE-B scores were classified as low, moderate, and high in 206 (31.2%), 287 (43.5%), and 166 (25.3%) individuals, respectively. Among the 659 patients, 31 (4.7%) developed HCC, a higher frequency than reported in PAGE-B score validation studies from other countries. Of these patients, 29 were male, with a mean age of 57.4±12.6 years and lower platelet levels (<200,000 10³/mL). Patients who developed HCC had fibrosis stages: F0-F1:6 (19.3%); F2:2 (6.4%); F3:2 (6.4%); and F4:21 (67.7%). High-risk patients were treated with ETV (n=129, 32%) versus TDF (n=37, 14,4%), *P*<0.00.

**Conclusion:**

The PAGE-B score demonstrated, in the Brazilian population, a performance similar to that observed in studies with European and Asian populations in terms of sensitivity, specificity, and predictive values for HCC prediction. Based on these results, the PAGE-B score can be used in the Brazilian population to predict the risk of HCC.

## HIGHLIGHTS

•The PAGE-B score was validated in the Brazilian population to predict the development of hepatocellular carcinoma (HCC) in patients with chronic hepatitis B (HBV).

•A cohort study of 659 mono-infected HBV patients treated with antivirals for at least 3 years found that 31 (4.7%) developed HCC.

•High-risk patients (25.3% of the cohort) were predominantly male, with a mean age of 57.4 years, and had lower platelet counts (<200,000×103/mL).

•The PAGE-B score demonstrated 80% sensitivity, 55% specificity, an 8% positive predictive value, and a 98% negative predictive value for predicting HCC.

## RESUMO

**Contexto:**

A infecção pelo vírus da hepatite B (VHB) por ter uma evolução em forma crônica e grave, recomenda-se o uso de medicamentos antivirais, Tenofovir (TDF) e Entecavir (ETV) para reduzir a carga viral do VHB e prevenir risco de cirrose hepática e carcinoma hepatocelular (CHC). Para avaliar o risco de desenvolver CHC, o escore PAGE-B é simples e confiável e ainda não foi validado no Brasil.

**Objetivo:**

Validar o escore de risco PAGE-B para predição do desenvolvimento de CHC em portadores de VHB no Brasil; Analisar a associação entre o escore PAGE-B e variáveis demográficas, laboratoriais e de tratamento do VHB.

**Métodos:**

Um estudo de coorte observacional e retrospectivo. Amostra do estudo: 659 indivíduos com VHB crônica mono-infectados tratados com antivirais por no mínimo 3 anos em 2 centros de referência do Nordeste e Amazônia do Brasil. A pontuação do escore PAGE-B foi utilizada para analisar sua associação com sexo, idade e contagem de plaquetas e classificou-se o risco de cada paciente desenvolver CHC como baixo, moderado e alto.

**Resultados:**

O Escore PAGE-B teve média 12,77±5,63. O escore PAGE-B foi baixo, moderado e alto em 206 (31,2%), 287 (43,5%) e 166 (25,3%) indivíduos, respectivamente. Dentre os 659 pacientes, 31 (4,7%) desenvolveram CHC, uma frequência maior do que a relatada em estudos de validação de pontuação PAGE-B de outros países. Nestes pacientes, 29 eram maior no sexo masculino, idade média de 57,4±12,6, com níveis mais baixos de plaquetas (<200.000 10^3^/mL). Os pacientes que desenvolveram CHC tiveram grau de fibrose: (F0-F1) 6 (19,3%); (F2) 2 (6,4%); (F3) 2 (6,4%); e (F4) 21 (67,7%). Os pacientes de alto risco foram tratados com ETV n= 129 (32%) versus TNF: n= 37 (14,4%) *P*<0,001.

**Conclusão:**

O escore PAGE-B demonstrou, na população brasileira, desempenho semelhante ao observado em estudos com populações europeias e asiáticas em termos de sensibilidade, especificidade e valores preditivos para predição de CHC. Com base nesses resultados, o escore PAGE-B pode ser utilizado na população brasileira para predizer o risco de CHC.

## INTRODUCTION

Hepatitis is a liver disease characterized by a necroinflammatory process caused by various agents, including xenobiotics and viral infections. Hepatitis B virus (HBV) infection can clinically present in acute, chronic, or fulminant forms^1^, being a global public health issue that can progress to liver cirrhosis (LC) and hepatocellular carcinoma (HCC)^2^. HCC can be primarily prevented through vaccination, which protects against HBV infection^3^. HCC development in individuals with HBV is a multifactorial process involving interactions between host and environmental factors. Reported risk factors for HBV-related HCC include high viral load, sex, age, smoking, alcohol consumption, chemical carcinogens, hormonal factors, and genetic susceptibility^4^. HCC accounts for 70% to 85% of primary liver neoplasms and is the most frequent primary liver tumor^5^.

In 2022, an estimated 254 million people will be living with chronic hepatitis B worldwide, with 1.2 million new infections annually. In the same year, hepatitis B was responsible for approximately 1.2 million deaths, mainly due to liver cirrhosis and hepatocellular carcinoma (HCC), although only 10% of cases are currently detected^6^. In Brazil, it is estimated that approximately 1 million people live with hepatitis B, of whom 700,000 remain undiagnosed. By 2022, 264,000 cases had been identified, and 41,000 people were undergoing treatment^7^. Although Brazil has, in general, a low prevalence of hepatitis B virus (HBV) infection, certain areas, such as the Amazon region, have a higher number of carriers. Between 2000 and 2023, 36.8% of viral hepatitis cases reported in the Notifiable Diseases Information System (Sinan) were hepatitis B. The disease is the second leading cause of death among viral hepatitis cases in Brazil, accounting for 21.7% of related deaths between 2000 and 2022^8^.

With the new Guidelines for Hepatitis B, it is estimated that more than 50% of people with chronic infection by the virus will require treatment, according to the context and established eligibility criteria^6^. The use of licensed oral medications for treatment, such as tenofovir (TDF), entecavir (ETV), and more recently, tenofovir alafenamide (TAF), is recommended, as these are considered the most potent drugs for suppressing HBV^6^ ^,^ ^9^. The quantification of serum HBV DNA levels (viral load) is used as a criterion to evaluate antiviral treatment in these patients^4^. Liver biopsy has been the gold standard for assessing liver fibrosis and its prognosis, but it is not performed on all patients with chronic hepatitis B due to its invasive nature and associated risks^10^. This method has been replaced by non-invasive approaches, such as liver elastography, which measures liver stiffness.

Recently, risk scores have been developed and validated for predicting HCC in cohort studies involving Asian and Caucasian patients, with the PAGE-B score (Platelet, Age, and Gender-HBV) being one of the most used. The PAGE-B score uses the variables platelet count, age, and gender of chronic hepatitis B carriers undergoing antiviral therapy and has demonstrated a high concordance index for predicting HCC development in Asian and Caucasian patients in Europe. However, the PAGE-B score has not yet been validated for the Brazilian population^11^. Therefore, the primary objective of this study is to validate the PAGE-B risk score for HCC development in HBV carriers in Brazil, analyzing the association of the PAGE-B score with demographic, laboratory, and histological host variables.

## METHODS

### Type of study

This study validates the PAGE-B score through a retrospective review of medical records from a historical cohort of chronic hepatitis B patients monitored at the Gastro-Hepatology Service of Professor Edgard Santos University Hospital (HUPES) and the Viral Hepatitis Outpatient Clinic at the Tropical Medicine Center of Rondônia (CEMETRON).

### Study population

Patients with chronic hepatitis B monoinfection in treatment with oral antivirals TDF or ETV for a minimum duration of 36 months. This study included a sample of 659 individuals monitored at two reference centers over 5 years. At the Gastro-hepatology Service of the University Hospital of the Federal University of Bahia, located in the state of Bahia, 118 patients were included, while 541 patients were from the Viral Hepatitis Outpatient Clinic at the Tropical Medicine Center of Rondônia, in the state of Rondônia.

The inclusion criteria were patients of both sexes with monoinfection, aged ≥18 years, undergoing treatment with TDF (tenofovir 300 milligrams (mg) per day) or ETV (entecavir 0.5 mg per day) for a minimum period of 36 months. The exclusion criteria were individuals without treatment, age <18 years, decompensated cirrhosis, a previous diagnosis of hepatocellular carcinoma (HCC) before starting treatment or within the first six months of therapy, a history of liver transplantation and/or patients on a liver transplant waiting list, and coinfections with hepatitis D, hepatitis C, or human immunodeficiency virus (HIV).

### Ethical aspects

This study was approved by the Research Ethics Committee (REC) of Professor Edgard Santos University Hospital at the Federal University of Bahia. It adhered to all standards for good practices in clinical research, with the Certificate of Ethical Appreciation Presentation (CAAE): 13814419.6.1001.0049.

### Variables collected for the study

Sociodemographic variables: Age ≥18 years; sex; race (white, mixed-race, black, Asian, Indigenous, or unknown); body mass index (BMI); and reference center.

Virus-related variables: HBeAg (Hepatitis B e Antigen) and HBV-DNA (Hepatitis B Virus DNA): ≤2,000 and >2,000 (IU/mL).

Histological variables: fibrosis stages were classified as follows - F0 - Absence of fibrosis; F1 - Portal fibrosis without septa; F2 - Portal fibrosis with few septa; F3 - Numerous septa without cirrhosis; and F4 - Hepatic cirrhosis. The fibrosis stage was determined based on histopathological analysis of liver biopsy specimens, performed by experienced pathologists using standard histological criteria. Additionally, the Fibrosis-4 (FIB-4) index was calculated using the formula: FIB-4=(age×AST) / (platelets×√ALT). FIB-4 values <1.45 were indicative of mild fibrosis, >3.25 suggested advanced fibrosis, and intermediate values were considered indicative of moderate fibrosis. This index was used as a complementary, non-invasive parameter to assess fibrosis severity.

Diagnostic Variable - HCC: diagnosis was established based on radiological and laboratory criteria. Computed tomography (CT) and contrast-enhanced magnetic resonance imaging (MRI) were used to identify typical HCC patterns (arterial phase hyperenhancement and venous washout), following the Liver Imaging Reporting and Data System (LI-RADS) criteria, with a definitive diagnosis in cases classified as LI-RADS 5. Ultrasound and alpha-fetoprotein (AFP) measurement were used as complementary methods for screening and monitoring. In cases with inconclusive or atypical radiological patterns (LI-RADS 3 or 4), patients were followed up every six months with re-evaluation by imaging and AFP to confirm or exclude progression to HCC. Thus, liver biopsy was reserved only for cases where the diagnosis could not be confirmed by non-invasive methods. The PAGE-B score was used as a prognostic tool to stratify the risk of HCC development in patients with chronic hepatitis B undergoing high genetic barrier antiviral therapy, guiding surveillance and early detection.

Laboratory variables: platelets: ≥200,000 (/mm³) normal, <200,000 (/mm³) low; Alkaline phosphatase (ALP): >150; Gamma-glutamyl transferase (GGT): Women: Reference range (RR) 05-43 U/L and Men: RR 07-60 U/L (normal); AST levels: >37 U/L elevated, <37 U/L normal; ALT levels: >41 U/L elevated, <41 U/L normal; Alpha-fetoprotein (AFP): ≤8.1 normal, >8.1 elevated (ng/mL); Total bilirubin (TB) and ALBI Index (Formula): (log_10_ bilirubin×0.66)+(albumin×−0.085), where bilirubin is in μmol/L and albumin in g/L. ALBI score classification: Grade I: ≤-2.60 (lower mortality risk), Grade II: >-2.60 to ≤-1.39 (intermediate mortality risk) and Grade III: >-1.39 (higher mortality risk).

### PAGE-B risk score

The PAGE-B score was calculated, ranging from 0 to 25 points. The score is determined based on age: 16 to 29 years, 0 points; 30 to 39 years, 2 points; 40 to 49 years, 4 points; 50 to 59 years, 6 points; 60 to 69 years, 8 points; ≥70 years, 10 points. It is also based on gender: female, 0 points; male, 6 points. Additionally, it considers platelet count (thousand/mm³): ≥200,000, 0 points; 100,000 to 199,999, 6 points; <100,000, 9 points. In this study, the minimum age was considered 18 years, as the original^12^ study used 16 years as the minimum age. The PAGE-B scores were classified according to risk: low (≤9 points); moderate (10 to 17 points); and high (≥18 points).

### Statistical analysis

The Statistical Package for the Social Sciences (SPSS) version 18 was used. Descriptive statistics were presented with continuous variables expressed as means and standard deviations (SD) or medians and interquartile ranges (IQR). Categorical variables were described as proportions. For the comparison of continuous variables between groups, the Mann-Whitney test was applied, and for proportions, Pearson’s Chi-square test or Fisher’s exact test was used.

Analysis of Variance (ANOVA) was used to compare variances between the means of different groups. Following the ANOVA test, the Bonferroni post hoc test was applied. This multiple comparison test is conservative, meaning it is less prone to Type I errors (false positives) and has lower statistical power. It is best suited when the number of comparisons is low. The Omnibus test, a Chi-square test, compares the likelihood of the current model versus the null model (in this case, the intercept). Its significance value is *P*<0.05, indicating that the current model outperforms the null model. The Omnibus test evaluates the null hypothesis that all predictors are unrelated to the effect sizes.

Cumulative probabilities of HCC occurrence in individuals were estimated by using the Kaplan-Meier method, compared with the Log-Rank test, for the different risk classification levels of the PAGE-B score. The Cox proportional hazards regression model was used to estimate the effect of various variables on the risk of HCC occurrence. The validity of the proportionality assumption was verified through Schoenfeld’s residual analysis. Hazard ratios and 95% confidence intervals were described, and a *P*-value <0.05 was considered statistically significant.

Discrimination and calibration measures were assessed to evaluate the model’s predictive performance. Discrimination was evaluated using Harrell’s c-index, and the calibration curve was used to assess the agreement between the probability of an individual remaining free of HCC for five years, as predicted by the model, and the Kaplan-Meier estimate (observed probability). The Kaplan-Meier estimate and the standard error were determined for each quintile of predicted probabilities.

The Receiver Operating Characteristic (ROC) curve was used to evaluate the sensitivity and specificity of the PAGE-B score in identifying hepatocellular carcinoma, with the area under the curve (AUC) indicating the test’s overall accuracy. An AUC close to 1 reflects high accuracy, while an AUC near 0.5 suggests random performance. The analysis also assessed the score’s ability to predict HCC occurrence over time, providing key metrics such as sensitivity, specificity, positive predictive value (PPV), and negative predictive value (NPV). These parameters are crucial for determining the score’s clinical utility in predicting the likelihood of HCC. Furthermore, the ROC curve helps identify the optimal cutoff point, balancing sensitivity and specificity to improve diagnostic accuracy.

Survival rates were calculated using Kaplan-Meier survival analysis, and the accuracy in predicting HCC occurrence was evaluated using a time-dependent area under the ROC curve at all study time points. The PAGE-B score’s sensitivity, specificity, PPV, and NPV were also estimated.

## RESULTS

The study sample comprised 659 patients with chronic hepatitis B, 118 (17.9%) from Professor Edgard Santos University Hospital and 541 (82.1%) from the Center for Tropical Medicine of Rondônia. Of the 659 participants, 31 were diagnosed with HCC.

The mean age was 52.2±12.7 years (21-91), and 449 (68.1%) were male. At the end of the follow-up period, 110 patients (16.69%) developed liver cirrhosis. Of the patients, 403 (61.2%) had treatment experience with ETV and 256 (38.8%) with TDF. The average treatment duration was eight years (3-20 years). The dominant race in the study was mixed-race, with 285 (45.6%) patients, followed by Black patients with 196 (31.4%), White patients with 134 (21.4%), and Indigenous patients with 10 (1.6%). For 34 patients, race information was not available. This study used a self-declaration model from the Brazilian Institute of Geography and Statistics (IBGE). Regarding fibrosis stages based on the METAVIR scale, the following data were obtained: (F0-F1) 355 (53.86%), (F2) 135 (20.48%), (F3) 59 (8.95%), and (F4) 110 (16.69%). Based on the scoring parameters of the PAGE-B score, the study population was divided into three risk groups: low risk (n=206; 31.2%), moderate risk (n=287; 43.5%), and high risk (n=166; 25.3%). Most of the population (n=287; 43.5%) fell into the moderate-risk group. These results are shown in Table 1.

**TABLE 1:** Demographic and clinical characteristics of the hepatitis B population undergoing treatment with oral antivirals TDF or ETV for a minimum of 36 months, according to the PAGE-B risk score classification for hepatocellular carcinoma development.

		PAGE-B		***P*-value**
	Low risk	Moderate risk	High risk	
Study population, n(%)	206(31,2)	287(43,5)	166(25,3)	<0,001
Reference center, n(%)				
HUPES Complex, UFBA	32(27,2)	53(44,9)	33(27,9)	0,525
CEMETRON Hospital	174(32,1)	234(43,3)	133(24,6)	
Sex, n(%)				
Male	72(16,0)	212(47,2)	165(36,8)	<0,001
Female	134(63,8)	75(35,7)	1(0,50)	
Race, n(%)1				
White	34(25,4)	62(46,3)	38(28,3)	<0,001
Mixed-race	69(24,2)	124(43,5)	92(32,3)	
Black	90(46,0)	84(42,8)	22(11,2)	
Asian or indigenous	5(50,0)	4(40,0)	1(10,0)	
BMI, mean±SD	27,68±30,75	25,71±21,39	30,14±35,66	0,423
HBeAg, n(%)				
Positive, reactive	58(47,2)	39(31,7)	26(21,1)	
Negative, non-reactive	148(27,8)	245(45,9)	140(26,3)	<0,001
Inconclusive	0(0,0)	3(100,0)	0(0,0)	
Fibrosis stage, n(%)				
F0-F1	120(33,8)	178(50,2)	57(16,0)	
F2	55(40,7)	51(37,7)	29(21,6)	<0,001
F3	27(45,7)	20(34)	12(20,3)	
F4	4(3,6)	31(28,2)	75(68,2)	
Cirrhosis, n(%)				
Yes	4(3,6)	31(28,2)	75(68,2)	<0,001
No	202(36,8)	249(45,3)	98(17,9)	
Treatment duration, mean±SD	7,37±3,77	8,32±3,74	8,55±3,60	0,004
Antiviral treatment				
ETV, n(%)	97(24,1)	177(43,9)	129(32,0)	<0,001
TDF, n(%)	109(42,6)	110(43)	37(14,4)	
HBV-DNA (IU/mL), n(%)				
<=2.000 IU/mL	171(31,2)	251(45,8)	126(23,0)	0,007
>2.000 IU/mL	35(31,5)	36(32,4)	40(36,1)	
Occurrence of HCC				
Yes	3(9,7)	13(41,9)	15(48,4)	0,003
No	203(32,3)	274(43,7)	151(24)	

^1^In the race assessment, there were n=34 (5.1%) missing data. HUPES: Professor Edgard Santos University Hospital. CEMETRON: Rondônia Tropical Medicine Center. Aghbe: HBV “e” antigen. BMI: body mass index. HBV-DNA: hepatitis B virus DNA. SD: standard deviation. ETV: entecavir. TDF: tenofovir. HCC: hepatocellular carcinoma.

During the follow-up period with antiviral treatment, which had an average of 8.1±3.7 years, 31 (4.7%) patients developed HCC. These patients were predominantly male and had cirrhosis, lower platelet levels (<200,000 10³/mL), reduced albumin levels, and elevated alpha-fetoprotein (AFP) levels. The most commonly used antiviral treatment was ETV, administered to 23 patients (5.8%). In the univariate analysis, factors such as advanced age, male sex, low platelet count, presence of cirrhosis, and elevated AFP levels showed a significant association with the development of HCC (all with *P*<0.05). Comparative data between the groups with and without HCC can be observed in Table 2.

**TABLE 2:** Comparison of characteristics of patients with and without HCC, monoinfected with hepatitis B, and undergoing treatment with the oral antivirals TDF or ETV for a minimum of 36 months.

	Patients with HCC	Patients without HCC	***P*-value**
	n=31	n=628	
Age, mean±SD	57,4±12,6	51,9±12,6	<0,001
PAGE-B risk score, mean±SD	16,64±4,67	12,58±5,6	0,980
Male, n(%)	29(6,5)	420(93,5)	<0,001
Female, n(%)	2(1)	208(99)	
Liver cirrhosis	24(21,8)	86(78,2)	<0,001
Antiviral treatment			
ETV, n(%)	23(5,8)	374(94,2)	0,896
TDF, n(%)	8(3,1)	254(96,9)	
HBeAg positive	6(4,9)	117(95,1)	0,271
Biochemistry data, mean±SD			
ALT, mean±SD	48,5±32,6	35,5±33,7	0,890
ALT level, n(%)			
≤ 41 U/L normal	17(3,4)	488(96,6)	
> 41 U/L elevated	14(9,1)	140(90,9)	
AST, mean±SD	104,7±187,1	37,5±43,2	0,302
AST level, n(%)			
≤ 37 U/L normal	10(2,2)	456(97,8)	
> 37 U/L elevated	21(10,8)	172(89,2)	
Albumin (g/dL)	3,91±0,67	4,21±0,86	0,172
Total bilirubin (mg/mL)	1,17±0,89	0,99±2,84	0,608
Alpha-fetoprotein (ng/mL)	1.941,9±9.455,6	25,52±482,2	<0,001
Platelets (103/mL)	187,5±117,3	193,43±74,42	<0,001
Alkaline phosphatase (U/L)	204,7±182,4	102,46±60,38	0,618
Gamma-GT (U/L)	123,3±119,8	61,68±83,04	0,792
FIB-4, n (%)			
Mild fibrosis (<)1.45	5(1,6)	308(98,4)	
Intermediate fibrosis 1.45-3.25	9(4)	215(96)	<0,001
Advanced fibrosis (>) 3.25	17(14)	104(86)	
ALBI index, n(%)			
GRADE I ≤-2.60	30(4,7)	610(95,3)	0,279
GRADE II >-2.60 to ≤-1.39	1(16,7)	5(83,3)	
GRADE III >-1.39	0(0)	5(100)	

Aghbe: HBV “e” antigen. ETV: Entecavir. TDF: Tenofovir. ALT: alanine transaminase. GGT: gamma glutamyl transferase. AST: aspartate aminotransferase. SD: standard deviation. HCC: hepatocellular carcinoma. ALBI index: Albumin,Bilirubin. FIB-4: FIBROSIS-4.

It can also be observed in Table 3, where the PAGE-B score was evaluated, that there was a statistical difference demonstrated by the p-value for some biochemical variables, which are considered relevant for the prognosis and monitoring of HCC, according to the risk groups: male sex, advanced age, low serum platelet levels, elevated serum AFP levels, and elevated serum levels of aspartate aminotransferase (AST), gamma-glutamyl transferase (GGT), and alkaline phosphatase (ALP).

**TABLE 3:** Association between laboratory tests performed after starting treatment with TDF and ETV, with a minimum duration of ≥3 years, and risk classification by the PAGE-B score.

	Low risk	Moderate risk	High risk	***P*-value**
Biochemical data, **mean±SD**				
ALT, mean±SD	35,95±41,16	35,84±27,20	37,25±33,97	0,237
**ALT level, n(%)**				
≤ 41 U/L normal	166 (32,8)	217 (43)	122 (24,2)	0,237
> 41 U/L elevated	40(26)	70(45,4)	44(28,6)	
AST, mean±SD	36,55±46,96	40,63±55,96	46,07±77,88	0,002
**AST level, n(%)**				
≤ 37 U/L normal	162(34,8)	201(43,1)	103 (22,1)	0,002
> 37 U/L elevated	44 (22,79)	86 (44,56)	63 (32,64)	
Albumin (g/dL)	4,23±0,53	4,20±0,86	4,11±0,73	0,201
Total bilirubin (mg/mL)	0,73±0,64	0,96±2,03	1,38±4,78	0,052
Alpha-fetoprotein (ng/mL)	2,85±1,70	13,42±2,92	3.570,3±11.673,9	<0,001
Alkaline phosphatase (U/L)	95,31±52,16	102,49±75,94	137,09,±108,19	<0,001
Gamma-GT (U/L)	51,26±64,33	60,84±85,38	88,75±102,43	<0,001
**FIB-4, n(%)**				
Mild fibrosis (<) 1,45	153(48,9)	144(46)	16(5,1)	
Intermediate fibrosis 1,45-3,25	34(15,1)	112(50)	78(34,9)	<0,001
Advanced fibrosis (>) 3,25	6(4,9)	33(27,3)	82(67,8)	
**ALBI index**				
GRADE I (≤)-2,60	201(31,4)	277 (43,3)	162(25,3)	0,062
GRADE II (>)-2,60 a (≤)-1,39	1(11,2)	4 (44,4)	4(44,4)	
GRADE III (>)-1,39	0(0)	5(100)	0(0)	

ALT: alanine transaminase. GGT: gamma glutamyl transferase. AST: aspartate aminotransferase. SD: standard deviation. ALBI index: albumin, bilirubin.

### Validation of PAGE-B risk classification

The mean PAGE-B score was 12.77±5.63. According to the PAGE-B score, 206 (31.2%), 287 (43.5%), and 166 (25.3%) patients were classified as low risk, moderate risk, and high risk for HCC development, respectively. Among these, 4 (3.6%), 31 (28.2%), and 75 (68.2%) patients had cirrhosis in the low-, moderate-, and high-risk groups, respectively (*P*<0.001). Three (9.7%), 13 (41.9%), and 15 (48.4%) patients developed HCC in the low-, moderate-, and high-risk groups, respectively (*P*<0.05).

Among the 31 patients who developed HCC, the stages of fibrosis were distributed as follows: F0-F1, 6 (19.4%); F2, 2 (6.5%); F3, 2 (6.5%); and F4, 21 (67.6%). Among the six patients with F0-F1 fibrosis treated with ETV, the average follow-up period was 11 years. Five (83.3%) were men from Rondônia, while one (16.7%) was from Bahia. Additionally, five (83.3%) had no family history of Hepatitis B, cirrhosis, or HCC. Regarding viral load, two patients (33.3%) had levels above 2,000 IU/mL, while four (66.7%) had levels equal to or below this threshold. Overall, patients classified as high-risk and those who developed HCC presented with more advanced stages of fibrosis.

**FIGURE 1:**
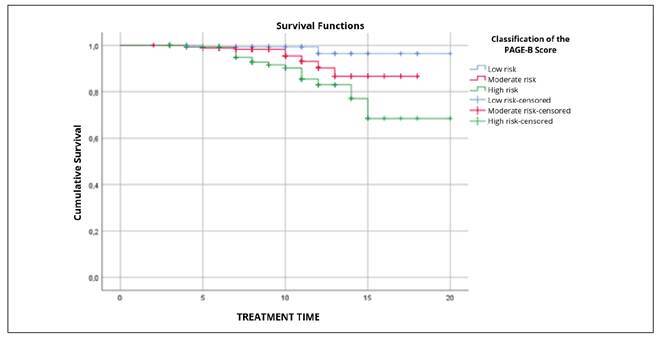
Survival Functions.

The mean follow-up and treatment time was 8.1±3.7 years, with a median of 7 [Interquartile Range: 5-11]. Using the Log Rank test, differences in the incidence of HCC were identified between the low, moderate, and high-risk groups [X2 (2)=11.095, *P*<0.004]. The incidence curves can be seen in Figure 1.

Low risk: blue line. Moderate risk: red line. High risk: green line. Low risk censoring: blue “+” symbol. Moderate risk censoring: red “+” symbol. High risk censoring: green “+” symbol.

Therefore, Cox regression with multivariate analysis was performed to verify the prediction of HCC according to the classification of the PAGE-B score. The Omnibus test [x2 (2)=11.617, *P*<0.003] demonstrated the adequacy of the models. Table 4 presents the main results of the analysis, showing that individuals classified as high risk on the PAGE-B score have a 7.43 times higher risk of developing HCC compared to those classified as low risk.

**TABLE 4:** Cox proportional hazard model for the PAGE-B score in patients undergoing treatment with oral antivirals TDF or ETV for a minimum of 36 months at the two reference centers: Gastro-Hepatology Service of Professor Edgar Santos University Hospital (HUPES) and the Center for Tropical Medicine of Rondônia (CEMETRON).

	B	DP	TWald test	gl	Exp(B)	***P*-value**	95,0%IC to Exp(B)	
							Lowest	Upper
PAGE-B Score Classification (Low risk)			9,031	2		0,011		
PAGE-B Score Classification (Moderate risk)	1,280	0,760	2,840	1	3,598	0,092	0,812	15,954
PAGE-B Score Classification (High risk)	2,006	0,750	7,142	1	7,430	0,008	1,707	32,344

DP: standard deviation. IC: confidence interval. Exp(B): hazard ratio. Gl: degrees of freedom.

**FIGURE 2:**
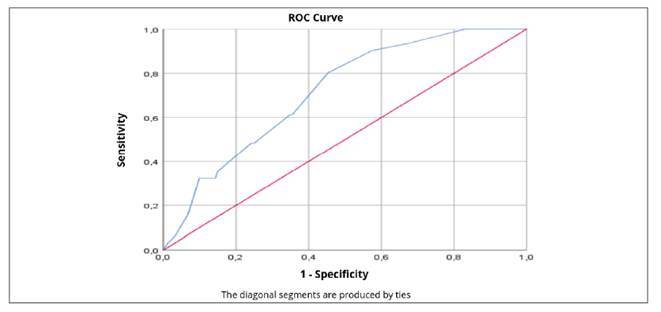
ROC curve for the PAGE-B risk score of patients monoinfected with the Hepatitis B virus, coming from the Gastro-Hepatology Service of Professor Edgar Santos University Hospital (HUPES) and the Rondônia Tropical Medicine Center (CEMETRON), between 2012 and 2018.

The ROC curve results validate the PAGE-B score, as shown in Figure 2. The results showed a statistically significant curve: AUC=0.715, SE=0.004, *P*<0.001, 95%CI 0.637-0.793. These findings indicate that, when chosen randomly, 71.5% of HCC cases had higher scores than cases without the disease. These findings suggest that the PAGE-B score has good discriminative ability to predict the risk of hepatocellular carcinoma (HCC), as, when randomly selecting an HCC case and a non-disease case, the PAGE-B score presented a higher score in the HCC group 71.5% of the time. The cutoff point that maximized the sensitivity and specificity of PAGE-B was <12.5. Supporting this result, there is an increased likelihood of individuals developing HCC if the cutoff point is >12, with a sensitivity of 80% and a specificity of 55% (similar results to those of Costa et al.^13^). The PPV was 8%, and the NPV was 98%.

## DISCUSSION

Among the current scoring systems, PAGE-B is the only one that does not include cirrhosis as a parameter. Cirrhosis is the main risk factor for HCC, with an annual rate of 2.5% to 4% in cirrhotic patients^14^. Considered critical for HCC development, cirrhosis is characterized by hepatic fibrosis and nodular regeneration, representing a premalignant condition^15^. In Western countries such as Brazil, 70% to 80% of HCC cases are associated with cirrhosis secondary to chronic infection with hepatitis B or C viruses^16^. In this study, the PAGE-B risk classification showed that 4 cases (3.6%) were at low risk, 31 (28.1%) were at moderate risk, and 75 (68.1%) were at high risk. Comparatively, Gokcen et al.^14^ identified 19 (6.8%) as low risk, 78 (22.9%) as moderate risk, and 64 (53.3%) as high risk (*P*<0.001). Papatheodoridis et al.^12^ reported 12 (3.9%), 105 (18%), and 144 (40.9%) in the same groups.

These findings highlight the importance of fibrosis assessment in the context of HCC risk. In this study, fibrosis was classified on a scale from F0 (no fibrosis) to F4 (cirrhosis), and stage F4 was predominant in the high-risk group, accounting for 68.2% of the cases, with statistical significance (*P*<0.001). It was observed that the number of patients in the high-risk group was 18 times greater than in the low-risk group and 2.4 times greater than in the intermediate-risk group. The Fibrosis-4 (FIB-4) index, a non-invasive method that evaluates liver fibrosis or cirrhosis, proved to be a valuable marker for monitoring patients with chronic hepatitis. FIB-4 was calculated using the formula: FIB-4 = (age × AST) / (platelets × √ALT), with values <1.45 indicating mild fibrosis, >3.25 suggesting advanced fibrosis, and intermediate values reflecting moderate fibrosis^17^.

Although fibrosis assessment demonstrated a strong correlation with the risk of HCC development, the data from this study indicate that the mean FIB-4 values for patients with HCC (M=4.9) and without HCC (M=2.45) showed statistical significance (*P*=0.0019). This suggests that while the FIB-4 method is a valuable indicator of fibrosis severity, it cannot reliably predict whether patients will develop HCC. However, patients with HCC exhibited a higher degree of fibrosis, as evidenced by the results, with the mean FIB-4 being higher compared to patients without HCC. This reinforces the conclusion that HCC patients present with a more advanced degree of fibrosis, consistent with expectations for this group.

High-risk patients presented only 5% with mild fibrosis, indicating that the PAGE-B score signals an increased risk even in these cases. It was observed that FIB-4 was elevated in patients with HCC and those without HCC. As risk increases, fibrosis also worsens, supported by FIB-4 findings, reinforcing the detection of advanced fibrosis obtained through other methods such as FibroScan and biopsy. FIB-4 demonstrated an association with high risk and good concordance among low- and intermediate-risk groups regarding mild fibrosis. HCC prediction is more accurate in cases of advanced fibrosis, making FIB-4 a valuable indicator. Thus, the PAGE-B score proved to be an effective marker for fibrosis and a reliable predictor of HCC.

Furthermore, in our study, most patients treated with first-line oral antivirals, ETV and TDF, demonstrated treatment adherence, which minimized the risk of viral resistance over time and its potential effects on HCC. Therefore, the PAGE-B score was developed and validated in patients treated with ETV and TDF^12^. Studies have identified patients with a lower cumulative incidence of HCC progression, particularly those undergoing treatment with nucleotide/nucleoside analogs^18^ ^,^ ^19^ ^,^ ^20^.

Regarding the treatment protocol with NAs, it was observed that the most commonly used medication in Brazil was entecavir (ETV), with 403 patients (61.2%) receiving this treatment. Particularly among high-risk patients with advanced fibrosis, 82 (67.8%) were predominantly treated with this medication. When comparing ETV and TDF, the ETV group showed a significantly higher percentage of high-risk patients, a statistically significant finding (*P*<0.001), likely because cirrhotic patients are preferentially treated with ETV^14^. However, subsequent studies identified patients with a cumulatively lower incidence of HCC development, especially those undergoing treatment with NAs. This uncertainty regarding residual HCC development in treated patients was addressed by the PAGE-B risk score, which primarily selects Caucasian patients at low risk for HCC (≤9 points) who did not develop HCC during stable therapy over a five-year follow-up. These data led the European Association for the Study of the Liver to recommend that, in patients with chronic hepatitis B classified as low risk by PAGE-B, the HCC surveillance strategy could be delayed. However, the risk of HCC is not eliminated, despite the efficacy of oral antiviral medications^13^.

Regarding patients diagnosed with HCC during treatment, 11 (35.5%) had detectable viremia >2,000 IU/mL HBV-DNA. Nonreactive HBeAg-negative status was higher than reactive HBeAg-positive results across all risk groups and showed statistical significance (*P*<0.001).

This study compared a low-endemicity region with a high-endemicity region, and a difference was observed. When patients from the two reference centers were compared using the PAGE-B score, there was no statistically significant difference between these two groups (*P*=0.525). Despite the fourfold difference in the population size between the Bahia and Rondônia regions, when the risk groups were compared, no significant differences were found that would invalidate this comparison. Thus, this validation can be extended to the Brazilian population.

In the treated patients, there was a predominance of mixed race in the moderate-risk group (43.5%) and the high-risk group (32.3%), compared to white race (46.3% and 28.3%, respectively), black race (42.8% and 11.2%, respectively), and Indigenous peoples (40% and 10%, respectively), with a statistically significant difference (*P*<0.001). In the study by Costa et al.^13^, a demographic questionnaire was used to compose the database, but information about the population’s race was neither described nor discussed in the article. The research focused on the Northeast region, specifically in the state of Maranhão, at the Liver Center of the University Hospital of the Federal University of Maranhão. As a result, there is insufficient data to compare racial characteristics with our study. The PAGE-B score has been extensively evaluated in Asian and Caucasian populations outside Asia, while most participants in this study were of mixed race, with this proportion remaining consistent across the two regions studied. Considering that the Brazilian population is highly diverse and that this study encompasses two reference centers in the country’s North and Northeast, this information is relevant for understanding the phenotypic characteristics of the population with chronic hepatitis B.

Concerning sex, the intermediate-risk and high-risk groups included 212 (47.2%) and 165 (36.8%) male patients, respectively, which was statistically significant (*P*<0.001), thereby validating the PAGE-B score. In this study, the variables age and male sex showed statistical significance (*P*<0.001) in the development of HCC. Studies indicate that the longer a person is infected with hepatitis B, the higher their risk of developing HCC. The mean age±SD of the group of patients who developed HCC during the follow-up period was 57.4±12.6 years, while for those who did not progress to HCC, it was 51.9±12.6 years, also showing statistical significance (*P*<0.001).

Regarding tumor markers associated with HCC, alpha-fetoprotein stood out in our study, showing a mean±standard deviation of 25.52±482.2 in patients without HCC and 1,941.9±9,455.6 in patients with HCC, with a statistically significant difference (*P*<0.001). As for the PAGE-B risk groups, serum AFP levels were higher in the intermediate- and high-risk groups, also with a statistically significant difference. In the study by Gokcen et al.^14^, higher AFP levels were similarly observed in individuals with HCC compared to those who did not develop HCC (*P*<0.05).

Serum levels of GGT and ALP were higher in the high-risk group. The mean±SD for these variables was 88.75±102.43 and 137.09±108.19, respectively, according to the PAGE-B score, showing statistical significance (*P*<0.001). Elevated serum GGT levels in this group align with other studies demonstrating that significantly higher GGT levels are observed in HCC patients with poorly differentiated tumors compared to those with well and moderately differentiated tumors, as reported by Corti et al.^21^.

Regarding the AST variable, its levels were elevated with statistical significance (*P*<0.05) according to the PAGE-B score risk classification; however, AST did not show statistical significance when comparing patients with and without HCC. Thus, it is understood that the result found is not highly significant as an indicator of HCC but is valid for patients with chronic hepatitis B, as they may experience liver impairment during the progression of HBV. In our study, most high-risk patients already presented with liver cirrhosis and advanced fibrosis, with a mean±SD AST level of 46.07±77.88, compared to 36.55±46.96 in low-risk patients. These values tend to increase with the rise in risk level. These findings are detailed in Table 3.

In this sample, the proportion of patients classified according to the PAGE-B score in the low-risk group was 206 (31.2%), in the high-risk group 166 (25.4%), and in the moderate-risk group, the highest proportion was observed at 287 (43.5%), a statistically significant finding (*P*<0.001). In the study by Gokcen et al.^14^, the proportions for the low-, moderate-, and high-risk groups were 281 (37.9%), 341 (46%), and 120 (16.2%), respectively. Similarly, in the study by Papatheodoridis et al.^12^, the proportions were 312 (24.7%), 597 (47.2%), and 355 (28.1%), respectively (*P*<0.001). This indicates that moderate risk was the most prevalent category in the studies by Gokcen et al.^14^ and Papatheodoridis et al.^12^, a finding that was also confirmed in our sample.

PAGE-B scores <9 indicate no or possibly minimal risk of HCC within five years, while scores ≥9, and particularly >18, indicate an increased risk of HCC, requiring continuous surveillance and care^12^. In our study, the cutoff point that maximized the sensitivity and specificity of the PAGE-B score was <12.5, where we demonstrated a good percentage similar to the findings of Costa et al.^13^.

It was also considered that the category of high-risk patients included those with chronic hepatitis B undergoing treatment with AN. When compared with the study by Costa et al.^13^, the cutoff point was the same (≥11), thus identifying a good sensitivity of the PAGE-B score in predicting the risk of developing HCC over a 5-year follow-up. This suggests that patients with less than 11 points on the PAGE-B score might require a longer follow-up than traditionally performed^13^. In the study by Gokcen et al.^14^, for a cutoff value of the PAGE-B score ≤9, similar reliability of the PAGE-B score in predicting the development of HCC was observed compared to a cutoff value of ≥10 in the study by Papatheodoridis et al.^12^. It was considered that the lower rates of HCC might be related to the relatively low total number of patients and the number of patients who completed the 5-year follow-up^14^.

The PAGE-B score in our study demonstrated 80% sensitivity, 55% specificity, 8% positive predictive value (PPV), and 98% negative predictive value (NPV) for predicting HCC in the validation datasets. These findings were similar to the results reported by Costa et al.^13^. In the study by Gokcen et al.^14^, the sensitivity, specificity, PPV, and NPV for HCC prediction were 96.2%, 39.1%, 5.4%, and 99.6%, respectively. Comparatively, in the study by Papatheodoridis et al.^12^, these values were 100%, 19.6%, 10.6%, and 100%, respectively. Our study’s results agree with those of these studies, making it possible to validate the PAGE-B risk score in the Brazilian population. Therefore, if these findings are confirmed in other cohorts, patients with chronic hepatitis B treated with ETV or TDF, who belong to the low-risk group according to the PAGE-B score, may avoid a late prognosis of HCC with a safety margin.

In patients with hepatitis B, it is recommended to perform ultrasonography and measure alpha-fetoprotein levels every six months. However, limited access to ultrasonography through the Brazilian Unified Health System (SUS) poses a significant challenge. Extending the interval between exams to one year may be a viable alternative for low-risk patients. This study aims to improve the follow-up of these patients within the SUS by proposing an approach that simplifies monitoring and enables healthcare professionals to adopt practical and accessible methods from the outset.

According to this study, the PAGE-B risk score provides a simple and easy-to-use tool for healthcare providers managing patients with hepatitis B. It effectively estimates the risk of developing hepatocellular carcinoma (HCC) in an innovative and cost-efficient manner, offering an accessible and valuable solution for the Brazilian healthcare system.

## CONCLUSION

The PAGE-B score, based on age, sex, and platelet count, has proven to be a simple and reliable tool for predicting the risk of hepatocellular carcinoma (HCC) during the first five years of therapy with entecavir (ETV) or tenofovir (TDF) in patients with chronic hepatitis B. Its validation showed good predictive performance (80% sensitivity and 55% specificity for high-risk patients), reinforcing its clinical utility. This stratification allows for more targeted clinical management and may optimize surveillance through non-invasive methods. However, HCC diagnosis confirmation still relies on imaging exams and, when necessary, biopsy and molecular tests, especially in cases with inconclusive or atypical findings.

## Data Availability

Data-available-upon-request
